# Transcriptomic Insights Into the Immune Repertoire of an Antarctic Sponge

**DOI:** 10.1002/ece3.72786

**Published:** 2025-12-22

**Authors:** Leslie K. Daille, Mario Moreno‐Pino, Eduardo Hajdu, Nicole Trefault

**Affiliations:** ^1^ Centro GEMA‐Genómica, Ecología & Medio Ambiente Universidad Mayor Santiago Chile; ^2^ Millennium Nucleus in Marine Agronomy of Seaweed Holobionts (MASH) Puerto Montt Chile; ^3^ Museu Nacional, Universidade Federal do Rio de Janeiro Rio de Janeiro Rio de Janeiro Brazil; ^4^ FONDAP Center IDEAL – Dynamics of High Latitude Marine Ecosystem Valdivia Chile

**Keywords:** Antarctic sponges, immune response, RNA seq, transcriptome

## Abstract

Antarctic marine sponges are essential components of the benthic fauna, playing a crucial role both through their own biological activities and their symbiotic relationships with diverse microorganisms. Yet, the transcriptional repertoire and the immune responses associated with interactions with microorganisms in this unique environment still need to be fully understood. Here, we investigated the transcriptional repertoire underlying the immune system processes of the Antarctic sponge *Myxilla* (*Burtonanchora*) *lissostyla*. We generated a de novo transcriptome and functional annotation for *M.* (*B.*) *lissostyla*, collected during the austral summer of 2019, 2020, and 2021. Our findings revealed an extensive transcriptional repertoire with a high and consistent expression of constitutive transcripts across the years. Key pathways related to immune response and homeostasis were the most expressed in the Antarctic sponge transcriptome, and a diverse array of immune receptors highlights the wide host immune repertoire. Low microbial abundance sponges share a vast repertoire of immune receptors, and a predominance of membrane‐bound PRRs was detected mainly in *M*. (*B.*) *lissostyla*, indicating a broad range of receptors available for initial interactions and engagement with microorganisms. The functional repertoire unveiled here establishes baselines for assessing potential functional changes that may arise due to climate change.

## Graphical abstract

Antarctic marine sponges are essential components of the benthic fauna, playing a crucial role in structuring this ecosystem through their own biological activities and their symbiotic relationships with diverse microorganisms. Yet, the transcriptional repertoire and the immune responses associated with interactions with microorganisms in this unique environment is not fully understood. The transcriptomic insights into the immune repertoire of the Antarctic sponge *Myxilla* (*B.*) *lissostyla* indicate a broad range of receptors for initial interactions and engagement with microorganisms.

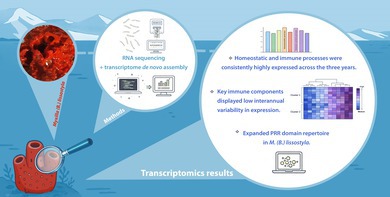

## 1 Introduction

Marine sponges, one of the most ancient metazoans, emerged approximately 600 million years ago and are a significant component of the benthic fauna in modern oceans (Taylor et al. 2007; Bell 2008). They play crucial ecological roles, including substrate modification, bentho‐pelagic coupling, and habitat provision for various marine organisms (Bell 2008). Sponges host a complex and diverse community of microorganisms, forming what is known as the sponge holobiont. This long‐standing symbiotic association provides a valuable model for studying fundamental host–microbe interactions in early‐diverging animals.

During feeding, sponges filter large volumes of seawater, interacting with a wide array of microbes. They are capable of distinguishing microorganisms ingested for nutrition and those maintained as symbionts (Wilkinson et al. 1984; Wehrl et al. 2007). The selective retention of specific symbionts suggests that these microorganisms have evolved strategies to evade host immune responses. In parallel, the sponge immune system can discriminate between harmful microorganisms and beneficial symbionts (Müller and Müller 2003; Germer et al. 2017).

Despite their structural simplicity, sponges exhibit a diverse array of innate immune mechanisms that are modulated by environmental factors and microbial interactions (Rathinam et al. 2024). Pattern recognition receptors (PRRs), key components of this system, play a central role in maintaining microbial symbiosis (Pita et al. 2016). PRRs recognize microbial‐associated molecular patterns (MAMPs) and pathogen‐associated molecular patterns (PAMPs), triggering immune responses that can promote homeostasis or an inflammatory response (Chu and Mazmanian 2013; Janeway Jr and Medzhitov 2002; Pita et al. 2018). PRRs detected in sponges include Toll‐like receptors (TLR), scavenger‐type cysteine‐rich receptor proteins (SRCR), NOD‐like receptors (NLR), and cytokine receptors (Wiens et al. 2007; Ryu et al. 2016; Srivastava et al. 2010; Germer et al. 2017; Pita et al. 2018; Schmittmann et al. 2021).

Given that the innate immune system is fundamental in maintaining the delicate balance between animal hosts and microbial symbionts, it is plausible that the environment modulates and drives the development of specialized adaptations. A very particular environment where sponges cover up to 80% of the seafloor is Antarctica. Antarctic sponges face extreme environmental conditions that demand unique adaptive strategies, with a notable proportion of endemic species (McClintock et al. 2005; Downey et al. 2012; Janusse and Downey 2014). The microbiomes of Antarctic sponges exhibit high diversity and functional specialization in processes such as nitrogen cycling, carbon fixation, and cold adaptation (Rodríguez‐Marconi et al. 2015; Moreno‐Pino et al. 2020, 2024). Despite their ecological importance, a significant gap exists in genomic and transcriptomic information for Antarctic sponges, which is crucial for understanding their symbiotic and immune mechanisms.

Available genomic data for the temperate sponges *Amphimedon queenslandica* and *Ephydatia muelleri* have provided important insights into sponge genome architecture and early animal evolution (Srivastava et al. 2010; Kenny et al. 2020). Transcriptomic studies further explored the gene expression dynamics across different life stages and environmental conditions, revealing regulatory mechanisms that underlie developmental and ecological responses (Conaco et al. 2012; Pérez‐Porro et al. 2013; Riesgo et al. 2014; Fernandez‐Valverde et al. 2015). Comparative analysis among *Xestospongia testudinaria* and *Petrosia ficiformis* has suggested a streamlined gene repertoire potentially reflecting adaptations associated with symbiosis or ecological specialization (Guzman and Conaco 2016). Consistently, Mediterranean sponges exhibit a notable immune receptor diversity and responsiveness to microbial cues, further supporting the role of host–microbe interactions in shaping sponge immune complexity (Pita et al. 2018). Building on these insights, subsequent analyses have suggested that the abundance of symbionts within sponge tissues may correlate with the diversity of immune receptors encoded in their genomes (Moitinho‐Silva et al. 2017). Multiple ecological and physiological differences between low‐microbial‐abundance (LMA) and high‐microbial‐abundance (HMA) sponges (Poppell et al. 2014; Lesser et al. 2022; Gan et al. 2024) also highlight the role of bacterial symbionts in maintaining host homeostasis.

In Antarctic species, transcriptomic analyses similarly indicate molecular adaptations to both environmental and microbial pressures. Studies on *Dendrilla antarctica* revealed signatures of selection in immune‐ and stress‐related genes, also suggesting molecular adaptations to environmental and microbial pressures (Leiva et al. 2019), while transcriptomic profiling of *Isodictya* sp. showed upregulation of TGF‐β, ubiquitin, and hedgehog signaling pathways in response to warming, indicating sensitivity to thermal stress (González‐Aravena et al. 2019).

Here, we generated a *de novo* transcriptome of the Antarctic sponge *Myxilla* (*Burtonanchora*) *lissostyla* Burton 1938 collected during the austral summers of 2019, 2020, and 2021. Our goal was to characterize the transcriptional profile of *M*. (*B*.) *lissostyla* and identify immune‐related expressed patterns potentially involved in host–microbe interactions in polar environments.

## 2 Materials and Methods

### 2.1 Sample Collection

Adult sponge samples of ten individuals of *M*. (*B*.) *lissostyla* were collected by scuba diving between 10 and 13 m in depth in December 2019, February 2020, December 2020, January 2021, and December 2021 from Chile Bay, Greenwich Island, South Shetlands, Antarctica. Sponge samples were cut with a sterile scalpel blade on the sampling site into 2 cm^3^ size pieces and immediately flash‐frozen in liquid nitrogen. These samples were preserved at −80°C until processing. Sub‐samples for taxonomic identification were preserved in 100% ethanol and kept at room temperature. The study was conducted under permits 1042/2019, 1043/2019, 802/2020, 803/2020, and 656/2021 granted by the Chilean Antarctic Institute (INACH).

### 2.2 Sponge Identification

Ethanol‐fixed subsamples from each sponge were used for taxonomic confirmation following standard protocols for the obtention of dissociated spicules and thick sections, as described in detail by Hajdu et al. (2011). Identification was performed after comparison with specialized literature and reference biological samples deposited in the MNRJ collection. Voucher fragments of sponge specimens were deposited in the Porifera collection of Museu Nacional/UFRJ under accession numbers MNRJ 24181, 24182, and 24201.

### 2.3 Phylogenetic Analysis

Cytochrome c oxidase gene (COX1) sequences were used for gene‐based taxonomic classification of the sponge. COX1 sequences identified in the transcriptome were compared against all COX1 sequences available for Porifera in the NCBI GenBank database at the time of analysis (*n* = 57). In addition, six sequences from other metazoans were included as outgroups, together with the two sequences assigned as COX1 by Gene Ontology (GO) database in this study, resulting in a final dataset of 65 amino acid sequences.

The evolutionary history was inferred using the Maximum Likelihood method and Le_Gascuel_2008 model (Le and Gascuel 2008) in MEGA11 (Tamura et al. 2021). A discrete Gamma distribution was used (5 categories) (+G, parameter = 0.6791). The final dataset comprised 452 informative positions and the initial phylogenetic trees were automatically generated using the Neighbor‐Joining and BioNJ algorithms. The tree topology with the highest log‐likelihood value was then selected for further analysis. The bootstrap consensus tree was generated from 250 replicates.

### 2.4 RNA Isolation

To maximize the repertoire captured in the assembled transcriptome, RNA was extracted from each sponge specimen. Sponge samples were cut into small pieces (5 mg). The fragments were mixed with 500 μL of Lysis Buffer from the mirVana RNA isolation kit (Invitrogen) and processed in a TissueLyser II (Qiagen) with 1 mm borosilicate solid‐glass beads in a 2 mL tube for 3 min at 30 Hz. The sponge macerates resulting from each sample were destined for separate subsequent steps for RNA isolation. RNA was extracted following the mirVana RNA isolation kit (Invitrogen) manufacturer protocol to obtain RNA > 200 nt. Extracted RNA was quantified using Qubit RNA HS Assay Kit, and its quality was evaluated with the Agilent Fragment Analyzer system. RNA from multiple individuals collected within the same austral summer (December 2019–February 2020, December 2020–January 2021, and December 2021) was subsequently pooled, resulting in three composite RNA samples representing the summers of 2019, 2020, and 2021, respectively.

### 2.5 RNA Sequencing, Filtering, and *de novo* Assembly

Enrichment of mRNA by capture of Poly‐A, rRNA library construction, and sequencing were performed at Novogene (USA), using an Illumina NovaSeq 6000 sequencing platform, with paired‐end sequencing (150 bp). Raw reads were filtered to remove adapters using Skewer v0.2.2 (Jiang et al. 2014). Low‐quality reads were removed using Trimmomatic v0.39, LEADING:5, TRAILING:5, SLIDINGWINDOW:4:15, MINLEN:30, Q > 33 (Bolger et al. 2014). These thresholds were chosen as standard values recommended in Trinity‐based *de novo* transcriptome assembly workflows, aiming to balance read retention with removal of low‐quality bases. Quality control was assessed with FastQC.

Further filtering involved the removal of ribosomal RNA using SortMeRNA v4.3.6 (Kopylova et al. 2012), using the database smr_v4.3_default_db.fasta. Reads were classified as bacteria, archaea, viruses, fungi, and microbial eukaryotes using Kaiju v1.9.2 (Menzel et al. 2016) in greedy‐5 mode using the nr_euk_2022‐03‐10 database. The remaining reads were used to generate a *de novo* transcriptome assembly with Trinity v2.8.5 (Grabherr et al. 2011; Haas et al. 2013) following the default parameters for paired‐end libraries. Individual assemblies were constructed following the same pipeline.

The quality of the transcriptome assemblies was evaluated using multiple approaches. Read representation was assessed by mapping back reads with Bowtie2 v2.3.5.1 (Langmead et al. 2009). The presence and completeness of protein‐coding genes were evaluated by comparison against the SwissProt database (release‐2022_05) using BLAST+ single best hit, with multiple high‐scoring segment pairs (HSPs) grouped to estimate alignment coverage (Camacho et al. 2009). Contig N50 values were obtained with Trinity toolkit utilities, and assembly quality metrics were further evaluated using TransRate v1.0.3 (Smith‐Unna et al. 2016). Completeness was assessed using Basic Universal Single Copy Orthologue (BUSCO) v5.4.4 against the Metazoa reference data (metazoa_odb10 dataset).

The transcript abundance was estimated using Salmon v0.13.1 (Patro et al. 2017) for each sample replicate to determine the expression of transcripts, measured as Transcripts Per Million. Cross‐sample normalized expression values were estimated using the TMM method (Robinson and Oshlack 2010), included in the Trinity toolkit utilities. After normalization, the total transcript isoforms were filtered, retaining isoforms with non‐zero normalized expression values for each year.

### 2.6 Functional Annotation

Assembled transcripts were annotated using Trinotate v3.2.2. (e‐values < 1e−5), which allowed the prediction of protein‐coding regions with Transdecoder v5.5.0 (Haas, BJ. https://github.com/TransDecoder/TransDecoder) and subsequently, it allows the homology search with BLAST+/SwissProt, the identification of protein domains using HMMER v3.3.2 and Pfam 35.0, and protein signal peptide using signalP v5.0b (Almagro Armenteros et al. 2019). Identifying genes related to sponge immunity was performed using GhostKOALA v2.0 (Kanehisa et al. 2016), KEGG decoder v1.3., and KEGG Mapper v5.0 (Kanehisa and Sato 2020; Kanehisa et al. 2022) to reconstruct KEGG pathways. When mapping genes into pathways, we categorized immune genes under the “Organismal systems: Immune system” category. In parallel, we used EggNOG 5.0 (Huerta‐Cepas et al. 2019) to annotate the transcript isoforms of metazoa origin. Gene Ontology (GO) and Enzyme Classification were used to determine the functions expressed by the sponge.

Additionally, a direct search based on previously reported immune signaling and effector domains (immunoglobulin‐like [Ig‐like] and fibronectin type III domains), cytoplasmic (NLRs through the presence of NLR domains, NACH domains, CARD domains, pyrin domains, and RIG‐I‐like receptor by the presence of RIG‐I domains), and membrane‐bound PRR domains (non‐canonical TLR was associated with the presence of the Toll‐interleukin receptor [TIR] domain and leucine‐rich repeats [LRR], SRCRs by the presence of SRCR domains, C‐type lectin receptors through the presence of lectin C‐type domains, GPCR associated with the presence of GPCR transmembrane domains) was performed using the PFAM annotation.

### 2.7 Comparative Analysis of Sponge Hosts Repertoire of PRRs and Immune‐Related Domains

Publicly available sponge transcriptomes under natural conditions *Amphimedon queenslandica* [SRR1513978], *Cymbastela stipitata* [SRR10127722], *Halisarca dujardinii* [ERR1143554], *Leucetta chagosensis* [SRR13528356], *Neopetrosia compacta* [SRR13528353], and *Oscarella lobularis* [ERR10149558] were filtered and assembled using the above detailed methodology used for the transcriptomes of this study. Information on the sponge sequences is detailed in Table S1.

Pfam domain searches were performed using HMMER v3.3.2 against the Pfam‐A 35.0 database. Hits were retained when they had a low e‐value and a bit score > 10, a permissive threshold chosen to maximize sensitivity to detect divergent domains that may be underrepresented in sponges. Domains were then classified into immune‐related categories based on previously reported associations. The comparative analysis across sponge species was performed at the presence/absence level, focusing on whether a given immune‐related domain was detected in each species, rather than on absolute counts. The specific Pfam identifiers used for each domain, together with the presence/absence values for each sponge, are provided in Table S2. A hierarchical clustering (hclust) analysis was performed using the complete linkage method and a Euclidean distance matrix to assess similarity in domain presence across species.

## 3 Results

### 3.1 Comprehensive Transcriptome Characterization of the Antarctic Sponge *Myxilla* Highlights a Diverse Gene Repertoire

**FIGURE 1:**
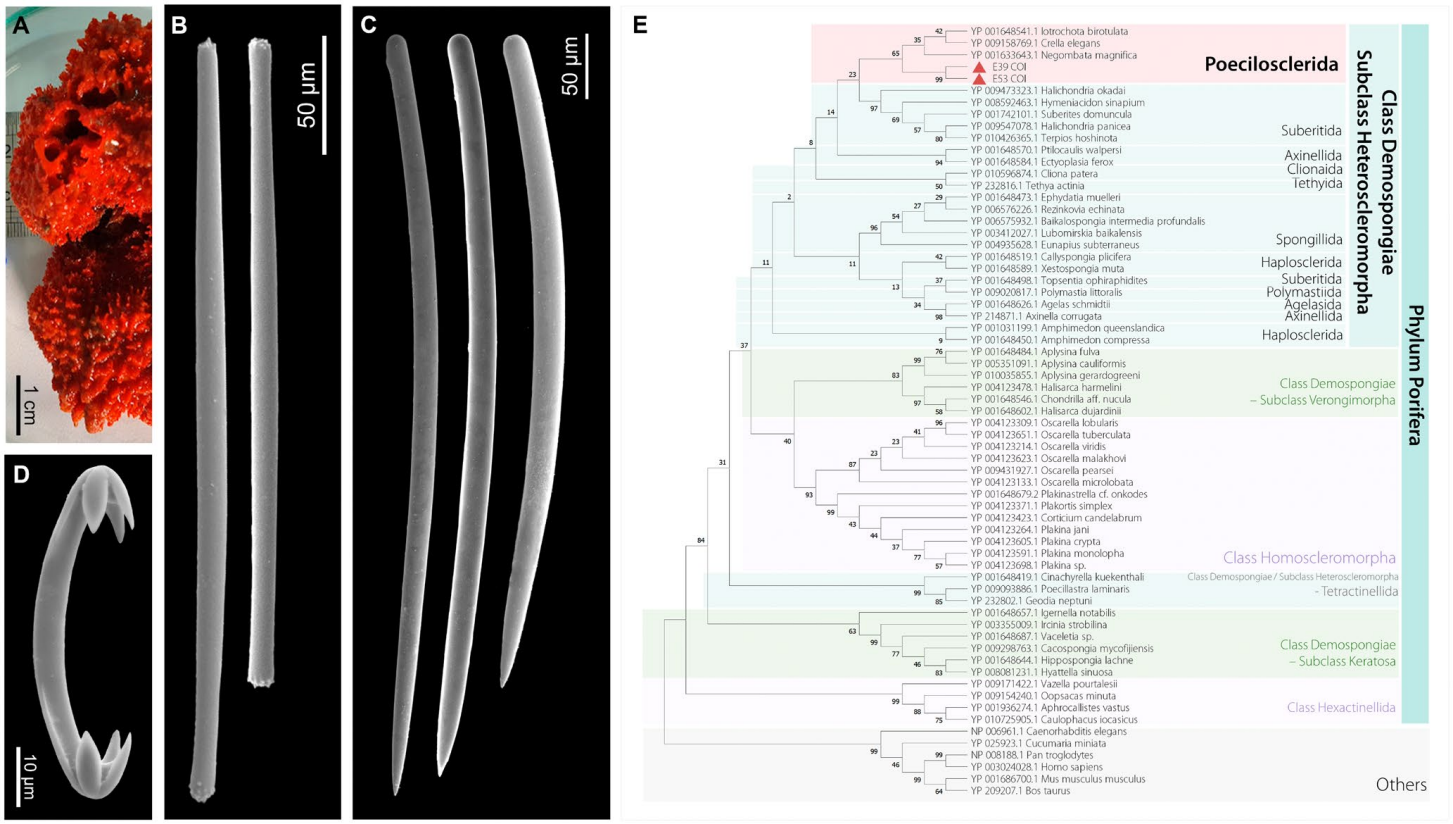
Taxonomic identification of *Myxilla* (*Burtonanchora*) *lissostyla* based on morphological characteristics and cytochrome c oxidase (COX1) protein sequences. (A) Macroscopic morphology of a representative sample. (B) Microscopic observation of subtylote. (C) Microscopic observation of megascleres (smooth style). (D) Microscopic observation of microscleres, representative anchorate isochela. (E) Maximum likelihood tree based on the COX1 protein. The bootstrap consensus tree was inferred from 250 replicates and comprised 65 amino acid sequences with 452 informative positions. Numbers above or below branches represent ML bootstrap support values. Sponge samples analyzed in this study are indicated with a red triangle and highlighted in light red shadow.

The sponges were taxonomically identified as *Myxilla* (*Burtonanchora*) *lissostyla* Burton, 1938, based on their spicule morphology. The materials studied here present spicules on the small size of the spectrum of micrometric variation reported for the species: styles 487–601 μm, subtylotes 244–325 μm, and anchorate isochelae 37–71 μm (Figure 1A–D). Geometrically, spicules are indistinguishable from those illustrated by Rios (2021), Rios (2007), and Schejter et al. (2024). The alignment of fragments of the COX1 gene confirmed the association with the order Poecilosclerida of the subclass Heteroscleromorpha of the Demospongiae (Figure 1E).

To characterize the transcriptome repertoire of the Antarctic sponge and identify the immune components involved in interactions with bacteria, we performed RNA‐sequencing of sponge samples collected over three different summer seasons from the South Shetland Islands. During this collection period, the seawater conditions at the collecting sites were relatively stable, with average temperatures of 0.86°C ± 0.44°C, salinity of 34.07 ± 0.04 PSU, and consistent nutrient levels (Table S2). This stability provided a reliable environmental context for evaluating the transcriptomic profiles.

RNA‐sequencing produced approximately 54 ± 0.4 million reads per library, with 40 million reads remaining after quality filtering and removal of ribosomal and microbial RNA (Table 1). The *de novo* reference transcriptome yielded 311,423 transcript isoforms corresponding to 173,675 genes, with an N50 of 1164 and a GC content of 47.7%. Individual transcriptomes had an average N50 of 1255 ± 20 bp and a GC content of 48.18% ± 0.05%, with an average contig length of 620 ± 22 nucleotides (Table 1).

**TABLE 1:** Statistics of the de novo transcriptome assemblies of the Antarctic sponge *Myxilla* (*Burtonanchora*) *lissostyla*.

Statistics	Reference	2019	2020	2021
Transcripts—Trinity isoforms	311,423	153,587	161,846	178,394
Genes—Trinity components	173,675	84,147	89,271	101,266
Average transcript length, nucleotides	583.69	644.35	627.81	624.61
Transcripts with ORFs	58,498	39,796	40,788	42,948
N50—all transcript contigs	1164	1278	1228	1260
N50—only longest isoform per “gene”	445	741	677	609
GC%	47.68	48.18	48.12	48.24
Total assembled bases, Mb	190.37	102.91	105.90	116.11

**FIGURE 2:**
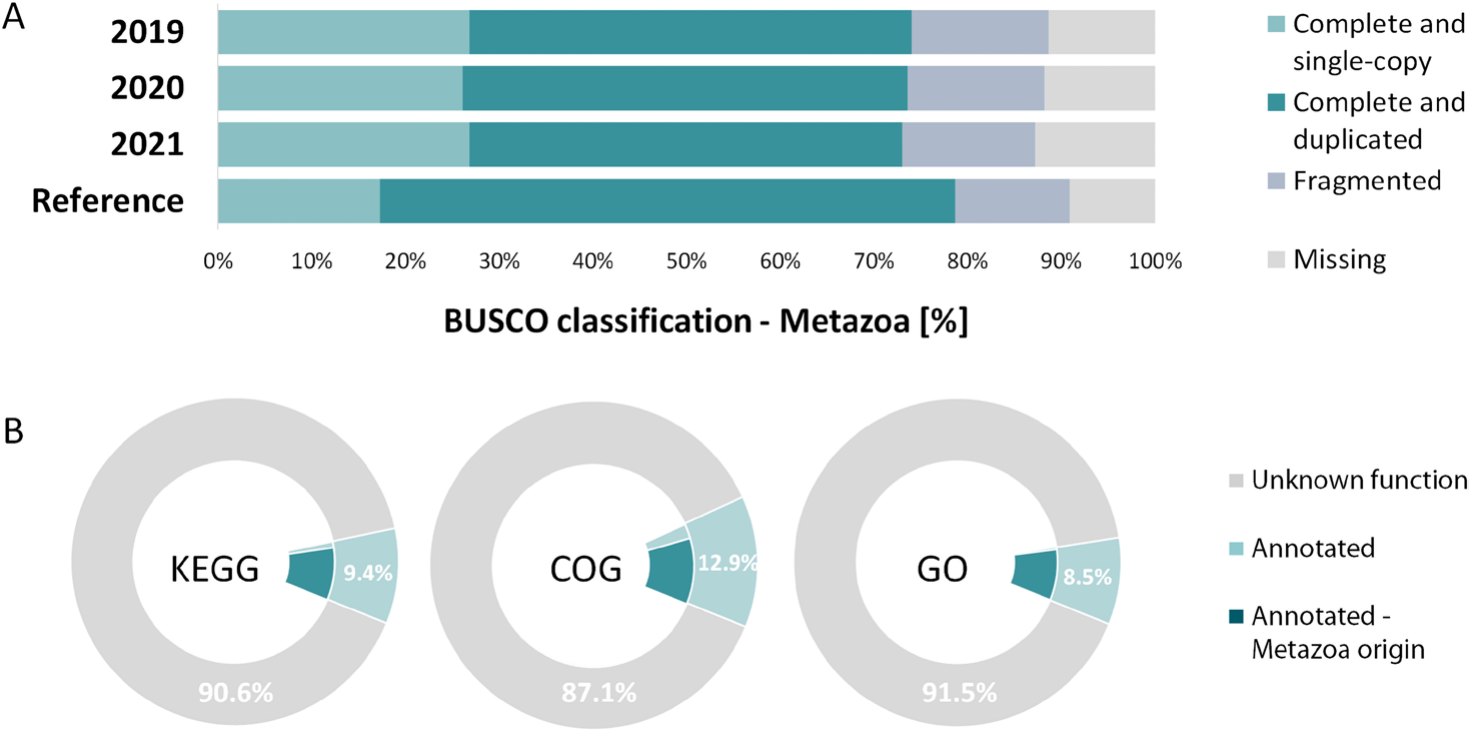
Classification of predicted transcripts isoforms. (A) Quantitative assessment of the completeness of the transcriptomes, based on the expected gene content compared to 954 genes of Metazoa. (B) Percentage of functional annotation using KEGG, Clusters of Orthologous Genes (COG), and Gene Ontology (GO) databases. The proportion of annotated transcripts as Metazoa is shown in dark shade.

The assemblies showed a high alignment rate (97.4%) against SwissProt, with 70.3% of transcripts containing ORF predictions. Notably, 89% of the 954 metazoan BUSCO genes were present, with 73.5% of these being complete models. This indicates a robust coverage of the codifying gene set and should allow for a comprehensive analysis of the transcriptional profile of an Antarctic sponge holobiont during the recent warm southern summers. Moreover, nearly two‐thirds of the complete models were represented by duplicated gene copies (Figure 2A).

After read normalization, we retained 116,045, 122,403, and 137,491 transcript isoforms per sample across the three summers, resulting in 237,938 unique transcript isoforms. On average, only 26% of these predicted transcripts showed similarity to known functions, while the remaining 74% were annotated as hypothetical proteins with unknown functions, highlighting a high novelty and an enormous functional and metabolic potential in the Antarctic sponge holobiont. Most of these annotations corresponded to Metazoa, as identified using KEGG, COG, and GO databases (Figure 2B).

### 3.2 Consistent Transcriptional Expression Highlights Steady Cellular and Metabolic Functions

**FIGURE 3:**
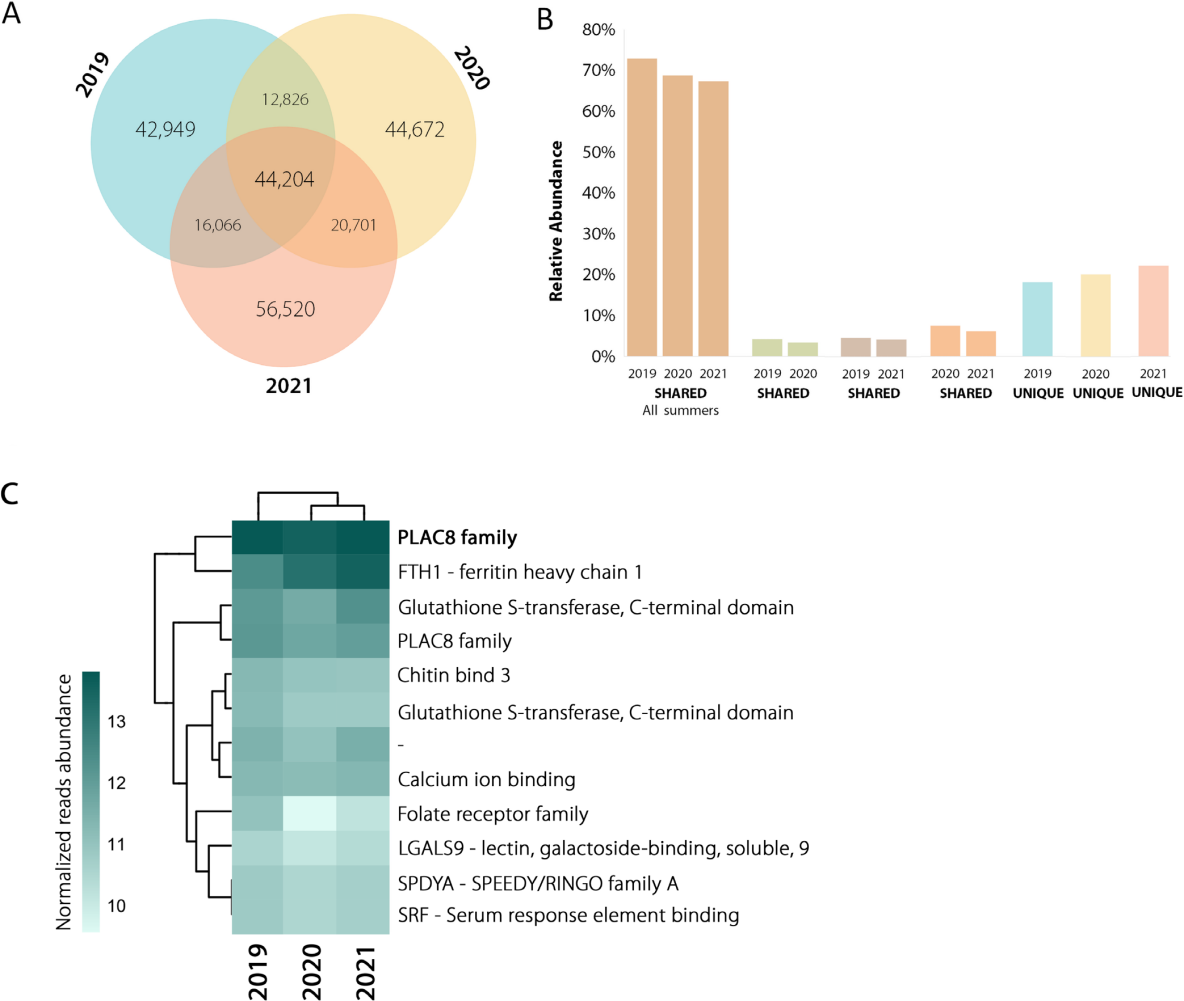
Transcripts expressed by *Myxilla* (*Burtonanchora*) *lissostyla* along the three summers. (A) Venn diagram showing the number of shared and unique transcripts expressed by the sponge during the three summers analyzed. (B) Relative expression of shared and unique transcripts. (C) Abundance of the top‐expressed transcripts across all years. Transcripts were annotated using the preferred names provided by eggNOG. Values correspond to TMM‐normalized reads and are clustered based on Bray–Curtis similarity.

A total of 44,204 transcripts, corresponding to approximately 19% of the assembled dataset, were shared among all analyzed samples (Figure 3A). Despite representing a small fraction of the transcriptome, these constitutive transcripts accounted for most reads: 73%, 69%, and 67% of the total for 2019, 2020, and 2021, respectively (Figure 3B). Additionally, the Bray–Curtis dissimilarity analysis of shared transcript expression showed that the transcriptomes of 2019, 2020, and 2021 were between 85% and 87% similar.

Functional annotation was obtained for approximately 26% of the transcriptome, a proportion typical for non‐model invertebrates but a limitation in functional inference. Within the annotated subset, the constitutive transcripts and plastic transcripts were mainly associated with cellular processes and signaling (40% ± 5%), followed by metabolism (19% ± 1.3%) and information storage and processing (18% ± 1.5%) (Figure S1).

Among the highly expressed core transcripts (Figure 3C), the most abundant (~11,900–14,300 reads) belonged to the placenta‐specific protein 8 (PLAC8) family, categorized under the “unknown function” (S) COG category. Other highly expressed transcripts were related to oxidoreductase activity, metal ion metabolism, glutathione S‐transferases, and lectin‐like domains, suggesting conserved roles in cellular homeostasis and redox balance (Table S3). A second, medium‐abundance group (4500–11,900 reads) comprised transcripts annotated within the inorganic ion transport and metabolism (P), general function prediction only (O), and S categories. Finally, a third group (1000–3000 reads) included transcripts involved in calcium ion binding, folate receptor activity, cyclin‐dependent protein regulation, and galactosidase binding, reflecting structural and regulatory diversity within the constitutive fraction. Overall, these results reveal a transcriptome dominated by a shared set of constitutive transcripts with essential metabolic and regulatory functions, accompanied by a smaller and potentially more plastic fraction that may contribute to physiological flexibility under the relatively stable environmental conditions observed at the sampling site (Table S4).

### 3.3 Key Pathways in Immune Response and Metabolism Dominate the Antarctic Sponge Transcriptome

**FIGURE 4:**
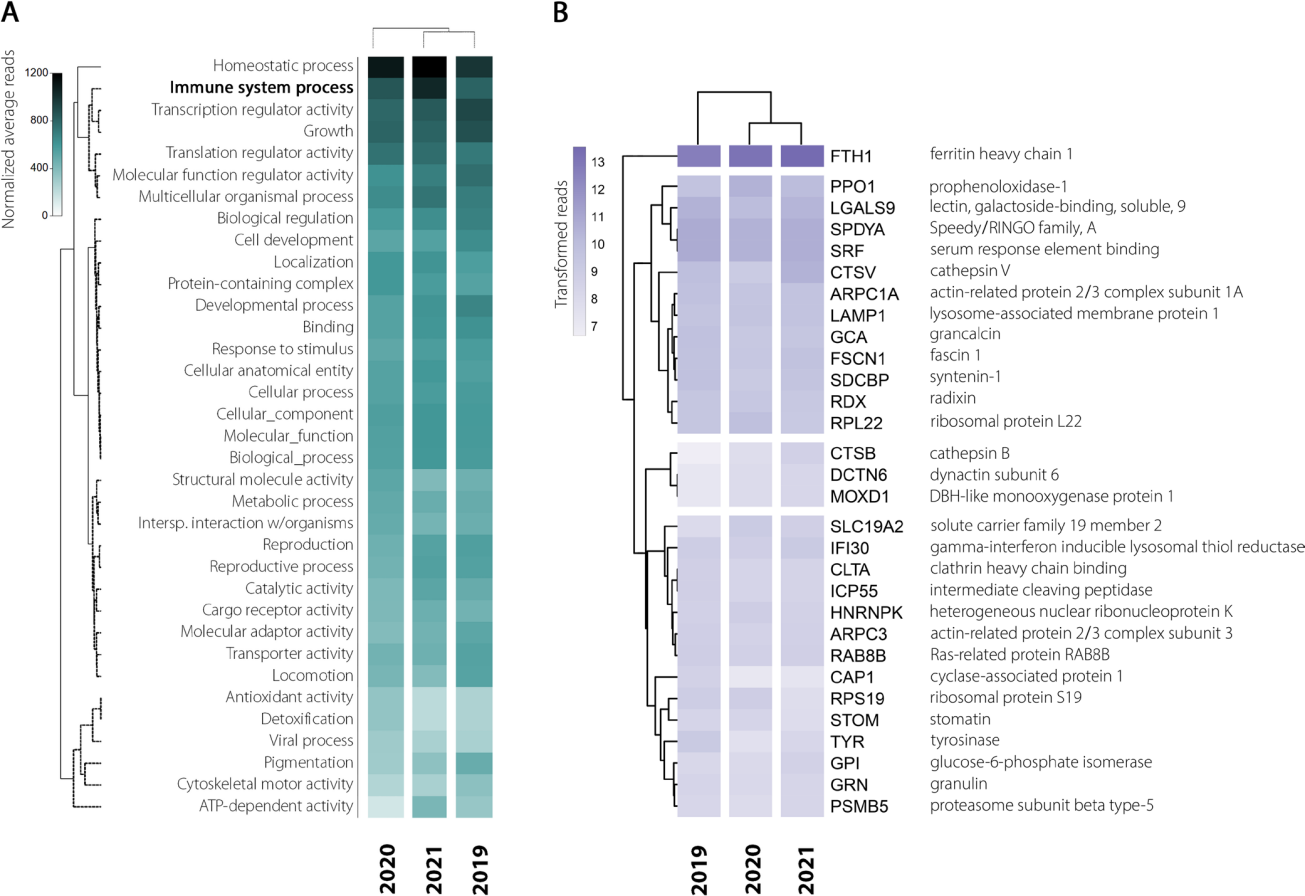
Most expressed transcripts in *Myxilla* (*Burtonanchora*) *lissostyla*. (A) The 100 most expressed transcripts were annotated using the Gene Ontology (GO) database and categorized according to GO level 2. The average read abundance of transcript isoforms from 2019, 2020, and 2021 transcriptomes was normalized using TMM. Expressed transcripts are clustered based on Bray–Curtis similarity. (B) Abundance of immune system process transcripts among the hundred most expressed in the transcriptome. The transcripts were annotated using the preferred name given by eggNOG. Values correspond to Log_2_(reads +1). Expressed transcripts are clustered based on Bray–Curtis similarity.

The top 100 most expressed transcripts showed a consistent expression profile across the summers (SIMPROF, 99% confidence level), particularly in pathways that regulate essential biological processes such as homeostasis, defense mechanisms, energy acquisition, growth, and interspecies interactions. Key functions included the transcripts related to localization, reproduction, response to stimuli, metabolic processes, pigmentation, detoxification, and viral processes (Figure 4A).

By examining the transcripts categorized under the immune system process ontology, we identified 30 distinct annotated genes among the most highly expressed transcripts (Figure 4B). These transcripts were grouped into two main expression clusters. The upper cluster included transcripts with the highest read counts, while the lower cluster contained transcripts with intermediate expression values. Expression levels were comparable among the three samples, with no marked year‐specific pattern. The most abundant transcripts across samples included FTH1, PPO1, LGALS9, SPDYA, SRF, CTSV, ARPC1A, LAMP1, GCA, FSCN1, SDCBP, RDX, and RPL22. A second group with lower normalized reads comprised CTSB, DCTN6, MOXD1, SLC19A2, IFI30, CLTA, ICP55, HNRNPK, ARPC3, RAB8B, CAP1, RPS19, STOM, TYR, GPI, GRN, and PSMB5. To explore potential intraspecific variability in immune gene expression, transcript abundances were standardized by Z‐score across the three samples (Figure S2). The resulting heatmap showed broadly comparable expression profiles among samples, with some transcripts (e.g., FTH1, IFI30, LGALS9, LAMP1) displaying relatively higher or lower expression in certain years, but overall very low levels, indicating subtle variation in expression intensity rather than major shifts in transcriptional patterns.

### 3.4 Low Microbial Abundance Sponges Share a Vast Repertoire of Immune Receptors

**FIGURE 5:**
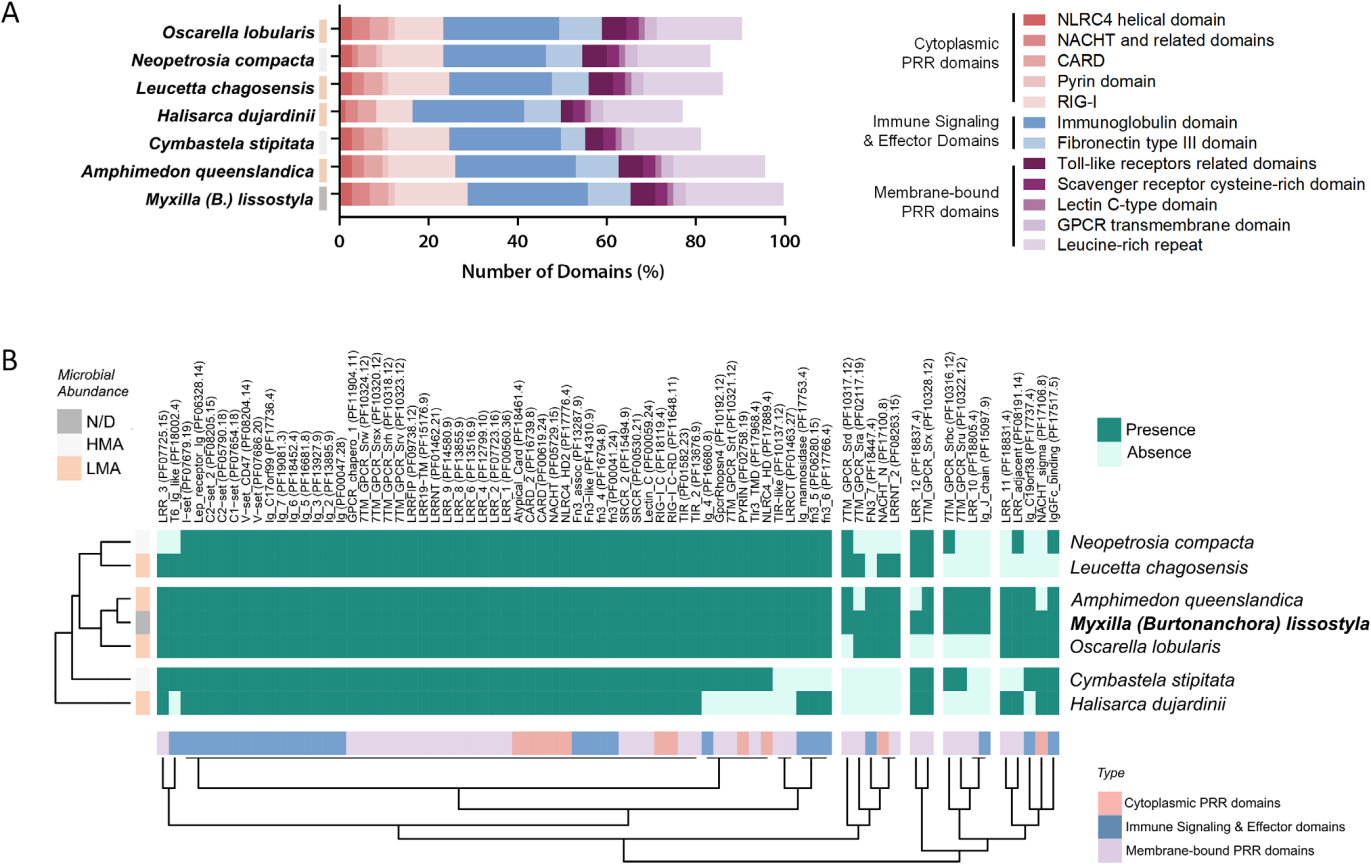
Prevalence of PRRs and immune‐related domains expressed in marine sponges. (A) Abundance of PRR categories and immune signaling and effector domains found in *Myxilla* (*Burtonanchora*) *lissostyla*, among other sponges transcriptomes. N/D (Gray), HMA (light gray), and LMA (light orange) classification is indicated using a colored key next to the respective sponge data. (B) Presence and absence distribution of the found domains in different sponges. HMA, high microbial abundance; LMA, low microbial abundance; N/D, not determined.

To determine whether the predominance of immune‐related functions is a common feature in sponge holobionts, a comparative analysis of protein domains related to immune receptors expressed in marine sponges was conducted, including membrane‐bound pattern recognition receptors (PRRs), cytoplasmic PRRs, and immunity‐related domains identified in the transcriptome of *M*. (*B*.) *lissostyla*. We examined their presence in the transcriptomes of the sponges *Amphimedon queenslandica, Cymbastela stipitata, Halisarca dujardinii, Leucetta chagosensis, Neopetrosia compacta*, and *Oscarella lobularis*. A total of 73 domains were found in *Myxilla*. Sponges with low microbial abundance (LMA) showed a homogeneous domain richness and exhibited a more extensive PRR repertoire compared to those with high microbial abundance (HMA), with the exception of *Halisarca dujardinii* (Figure 5A). No PRR domain was found exclusively in *M*. (*B*.) *lissostyla*; however, among those shared with other LMA sponges, we observed a predominance of membrane‐bound PRRs. These results indicate that *M*. (*B*.) *lissostyla* presents an expanded receptor repertoire for engaging with microorganisms as the first point of contact (Figure 5B).

Among the cytoplasmic PRR domains, we identified NOD‐like receptors (NLRs) associated domains, including NACHT domains (*n* = 3), CARD (Caspase recruitment domain, *n* = 3), Pyrin domain (*n* = 1), NLRC4 (*n* = 2), and RIG‐I‐like receptor domains. For membrane‐bound PRR domains (MEM), we found domains of the Toll‐like receptor family (TLR), including Toll/interleukin‐1 receptor homology domains, a nucleotide‐sensing TLR activated by double‐stranded RNA (Tlr3_TMD), and a C‐type lectin domain (*n* = 1), all of which were present in *M*. (*B*.) *lissostyla* and in most of the analyzed sponges (Figure 5B).

We identified 16 LRR domains that are typically involved in protein–protein interactions during microbial adhesion and the induction of immune responses. Additionally, we found several Scavenger receptor cysteine‐rich domains (SRCRs), including SRCR, SRCR_2, and Hepsin‐SRCR domains, which are known for their roles in pathogen recognition and clearance. Our assessment also revealed a diverse set of GPCR transmembrane domains, totaling 12. This group was one of the least prevalent among the sponges we evaluated. Finally, we identified several immune signaling and effector domains, including immunoglobulin‐like domains (*n* = 20) and Fibronectin type III domains (*n* = 7) (Figure 5B).

## 4 Discussion

This study presents a transcriptome analysis of the Antarctic sponge *M*. (*B*.) *lissostyla* collected over three consecutive summers from the South Shetland Islands, Antarctica. To the best of our knowledge, this is the third transcriptome of an Antarctic sponge available. The main objectives were to determine the transcriptomic repertoire of the sponge and to characterize the immune components that potentially interact with its bacterial partners.

Both morphological and molecular identification support the classification of our samples as *M*. (*B*.) *lissostyla*, which belongs to the order Poecilosclerida. Integrating both approaches is essential for reliable sponge classification, particularly given the limitations of current databases (Yang et al. 2017). Although there is no evidence for monophyly within the Myxillidae family (Redmond et al. 2013), our phylogenetic analysis based on mitochondrial COI sequence alignment supports this taxonomic assignment. Furthermore, recent molecular studies focused on the genus *Myxilla* suggest that it likely represents a polyphyletic assemblage, with species appearing in various positions on phylogenetic trees depending on the molecular markers used (Vargas et al. 2015; Rios and Cristobo 2014). This phylogenetic complexity emphasizes the importance of careful interpretation when comparing immune‐related genes across different *Myxilla* species, as evolutionary relationships may be more complicated than implied by classifications based solely on morphological characters.

We identified on average 164,609 transcript isoforms, corresponding to the expression of ~40,000 transcripts with ORF predictions, highlighting the complexity of the gene repertoire of *M*. (*B*.) *lissostyla* when compared to other sponges' coding sequence predictions (Steffen et al. 2023). The analysis of the hundred most expressed transcripts revealed an enrichment of immune‐annotated genes, all of them forming part of the constitutive transcripts shared across the analyzed years, suggesting that immune‐related processes may constitute a major component of the sponge's basal transcriptional activity (see Figure 4). This pattern supports the hypothesis of a baseline readiness in sponge tissues to respond to microbial challenge.

For instance, the detection of LGALS9 and GRN among the top‐expressed genes is consistent with the idea that lectin‐like molecules and growth/regenerative factors may serve dual roles in microbial recognition and maintaining tissue homeostasis (Pfeifer et al. 1993; Sun et al. 2022). In demosponges, lectins have been characterized as cell‐surface or secreted carbohydrate‐binding proteins implicated in microbial binding and aggregation, supporting their role as primitive pattern‐recognition receptors (Pfeifer et al. 1993). The co‐expression of multiple cytoskeletal regulators, such as ARPC1A, ARPC3, FSCN1, RDX, and regulatory elements like SRF, suggests that sponge cells may reorganize actin networks to facilitate particle uptake or phagocytosis. Indeed, in transcriptomic challenge experiments, sponges upregulate cytoskeletal and vesicle trafficking genes when exposed to microbial‐associated molecular patterns (Pita et al. 2018). The presence of lysosomal components (e.g., CTSB, CTSV, LAMP1, IFI30) among highly expressed genes further aligns with the hypothesis that internalized microorganisms are degraded intracellularly (Huynh et al. 2007). In *Suberites domuncula* , a MyD88‐dependent pathway is activated upon bacterial challenge, culminating in expression of antimicrobial effectors (Wiens et al. 2005). The expression of FTH1 may indicate engagement of iron sequestration or nutritional‐immune strategies, restricting microbial growth by limiting free iron (Ullah and Lang 2023). Concurrently, the detection of PSMB5 and STOM could reflect a need for proteostasis and membrane integrity under stress induced by immune activation. Genes like TYR, PPO1, and possibly MOXD1 raise a compelling hypothesis: that sponge tissues may engage melanin or phenoloxidase‐based defense or pigment‐based encapsulation, reminiscent of known melanization responses in demosponges during graft rejection (Wiens et al. 1998). Finally, the presence of SDCBP (syntenin‐1) and GCA could hint at intercellular signaling or Ca^2+^‐mediated coordination, perhaps enabling immune communication across sponge cell types (Perovic et al. 2001). Overall, the pattern strongly supports the idea that sponges possess a diverse innate immune toolkit comprising recognition, cytoskeletal remodeling, intracellular degradation, resource withholding, pigment‐based barriers, and cell–cell coordination, consistent with core principles of animal innate immunity. Future localization and challenge experiments are needed to test these observations directly.

We identified several transcripts containing domains typical of immune receptors, including multiple NLR‐like sequences with NACHT, CARD, and LRR motifs, suggesting potential capacity to recognize intracellular signals associated with infection or cellular stress. The similarity of this domain composition to that described in the LMA sponge *Stylissa carteri* (Ryu et al. 2016), and their lower representation in some HMA species (Germer et al. 2017), suggests that *M*. (*B*.) *lissostyla* likely follows an NLR configuration typical of LMA demosponges. However, given the limited resolution of transcriptomic data, this hypothesis requires confirmation through comparative genomic approaches.

We also detected transcripts encoding G protein–coupled receptors (GPCRs), which in other metazoans participate in transducing extracellular cues into intracellular signaling cascades (Krishnan et al. 2012; Dierking and Pita 2020). Although the roles of GPCRs in sponge immunity remain largely unexplored, studies in invertebrates such as *Caenorhabditis elegans* (Reboul and Ewbank 2016), *Drosophila melanogaster* (Ha et al. 2009), and cnidarian–dinoflagellate symbioses (Peng et al. 2010; Matthews et al. 2017) have linked these receptors to microbial sensing and homeostatic regulation. In Mediterranean sponges, similar GPCR‐associated signaling has been proposed to influence the stability of host–microbe associations (Pita et al. 2018). Therefore, the presence of diverse GPCR transcripts in *M*. (*B*.) *lissostyla* may reflect a conserved mechanism for mediating interactions with microbial partners or responding to environmental stimuli.

The co‐expression of these receptor domains together with apoptosis‐ and phagocytosis‐related genes supports a working hypothesis in which receptor activation may trigger immune‐like cascades, including programmed cell death and tissue remodeling, as previously observed in *Aplysina aerophoba* and *Dysidea avara* upon MAMP exposure (Pita et al. 2018). Considering that *M*. (*B*.) *lissostyla* is likely an LMA sponge, its PRR repertoire may be optimized for maintaining fewer but more specialized microbial associations, consistent with the ecological distinctions between LMA and HMA species (Gloeckner et al. 2014). The observed NLR diversity could enhance the capacity to discriminate among symbionts, contributing to the maintenance of stable yet dynamic host–microbe relationships. However, we acknowledge that these interpretations are based on transcriptomic evidence and that confirming the full extent of receptor diversity would require chromosome‐level genome assemblies and comparative genomic analyses across multiple sponge lineages.

Although this study provides valuable insight into the immune‐related transcriptional landscape of *M*. (*B*.) *lissostyla*, several limitations should be considered when interpreting the results. The analyses were based on a limited number of samples collected across different years without biological replication, which restricts the assessment of interindividual variability and statistical power. In addition, the use of transcriptomic data rather than complete genomic references may underestimate the representation of multicopy gene families or domain diversity. Therefore, the patterns described here should be viewed as descriptive and exploratory, serving as a framework for future studies integrating higher‐resolution genomic data and increased sample replication.

In summary, this study provides the first transcriptomic resource for *M*. (*B*.) *lissostyla*, highlighting the complexity of the host immune repertoire, which includes immune‐ and homeostasis‐related pathways and a diverse repertoire of PRRs and downstream immune effectors. Future studies should focus on experimentally validating these recognition and response elements to better understand microbial interactions with the host and how they modulate immune responses to maintain a stable yet adaptable microbiome. Comparative analyses with other Antarctic marine invertebrates could also provide broader insights into the evolutionary pressures driving immune complexity in polar ecosystems.

## Author Contributions

**Leslie K. Daille:** conceptualization (equal), formal analysis (equal), funding acquisition (equal), investigation (equal), methodology (equal), visualization (equal), writing – original draft (equal), writing – review and editing (equal). **Mario Moreno‐Pino:** investigation (supporting), methodology (supporting), writing – review and editing (supporting). **Eduardo Hajdu:** formal analysis (equal), methodology (equal), writing – review and editing (supporting). **Nicole Trefault:** conceptualization (equal), funding acquisition (lead), investigation (lead), project administration (lead), resources (lead), supervision (lead), writing – original draft (equal), writing – review and editing (equal).

## Funding

This work was supported by ANID ‐ Fondo Nacional de Desarrollo Científico y Tecnológico (3220873, 3210656, 1230758, 1190879) andFundação Carlos Chagas Filho de Amparo à Pesquisa do Estado do Rio de Janeiro (2233).

## Ethics Statement

All Antarctic field sampling was conducted in full compliance with the regulations of the Chilean Antarctic Institute (INACH) and national legislation, as well as with international agreements governing Antarctic research and the sustainable use of biodiversity.

## Conflicts of Interest

The authors declare no conflicts of interest.

## Supporting information


**Figure S1:** Functional composition of shared and unique transcripts in *Myxilla* (*Burtonanchora*) *lissostyla*. Relative abundance of functionally annotated transcripts across samples from 2019, 2020, and 2021, classified according to COG categories. Bars represent the proportional distribution of transcripts within the categories of Information storage and processing, cellular processes and signaling, metabolism, and poorly characterized functions. Shared and unique fractions are shown separately for each sampling period.

**Figure S2:** Heatmap of *Z*‐score–normalized expression values for immune‐annotated transcripts. Each row represents one of the 30 transcripts categorized under the Gene Ontology term immune system process. Expression values were transformed by Log_2_(reads +1) and were standardized by *Z*‐score across transcripts to highlight relative deviations from the mean expression level for each gene.

**Table S1:** Metadata and download links for publicly available sponge RNA‐Seq datasets. This table summarizes key information for transcriptomic datasets used in comparative analyses. Columns include species name, project, and sample identifiers (BioProject, SRA, and SRX accessions), sequencing strategy, instrument platform, library construction details, and condition descriptions. Download links for paired‐end reads (when applicable) are provided via EBI's FTP server.

**Table S2:** Pfam domains associated with immune‐related receptors across sponge species. This table summarizes the Pfam domains identified in *Myxilla* (*Burtonanchora*) *lissostyla* and in publicly available transcriptomes of *Amphimedon queenslandica* [SRR1513978], *Cymbastela stipitata* [SRR10127722], *Halisarca dujardinii* [ERR1143554], *Leucetta chagosensis* [SRR13528356], *Neopetrosia compacta* [SRR13528353], and *Oscarella lobularis* [ERR10149558]. Domains are associated into major receptor categories (TIR, RLR, CLR, SRCR, Fn, NLR, GPCR, Ig). For each domain, the Pfam identifier, domain description, and presence (1) or absence (0) in each species are reported.

**Table S3:** Environmental parameters measured at the sponge sampling sites. Physico‐chemical parameters measured during sampling at the South Shetland Islands, Antarctica. The table includes sampling date, geographic coordinates, depth, and seawater values for nutrients (${\mathrm{NO}}_{2}^{-}$, ${\mathrm{PO}}_{4}^{3-}$, ${\mathrm{SiO}}_{4}^{4-}$, ${\mathrm{NO}}_{3}^{-}$), salinity, temperature, and turbidity. Values are presented as means ± standard deviation where applicable.

**Table S4:** Annotated transcript isoforms of *Myxilla* (*Burtonanchora*) *lissostyla* across three consecutive years. This table lists annotated transcript isoforms with their corresponding COG category, best ortholog description, EC number, PFAM domains, and normalized abundance values for the years 2019, 2020, and 2021. The file is divided into three sheets based on expression levels: (i) Most_expressed_cluster: top‐expressed transcripts across all years; (ii) 50–1000 cluster: transcripts with intermediate abundance; 0–50 cluster: transcripts with the lowest abundance.

## Data Availability

Sequence data have been submitted to the GenBank database under accession number BioProject PRJNA1245495.
