# Supplementary figures and images for: Transcriptomic Insights Into the Immune Repertoire of an Antarctic Sponge
Source: Ecol Evol. 2025 Dec 22;15(12):e72786. doi: 10.1002/ece3.72786 (PMC12723187; doi:10.1002/ece3.72786)

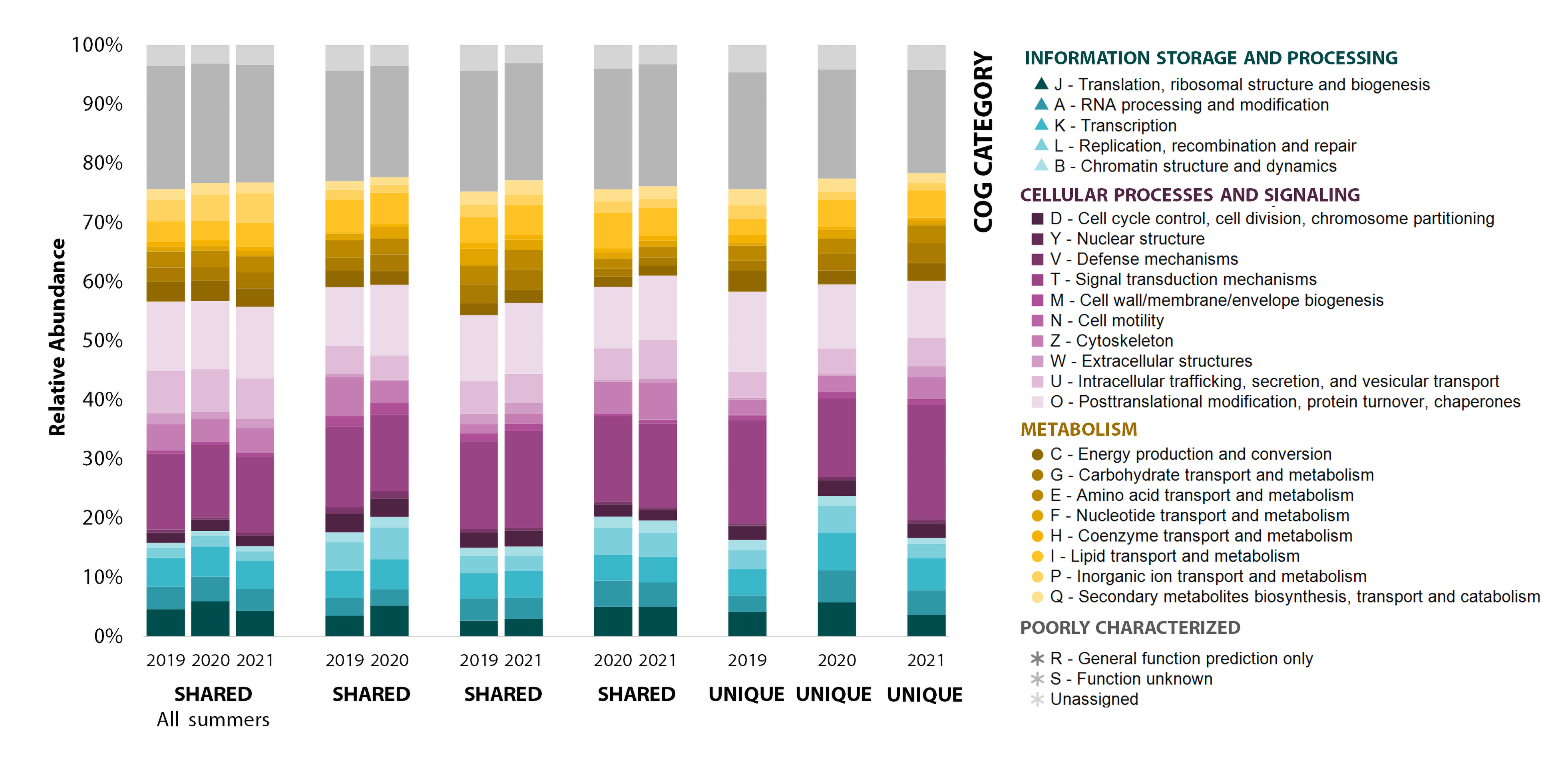

Supplement: Supplementary file 1 — Figure S1: Functional composition of shared and unique transcripts in Myxilla (Burtonanchora) lissostyla. Relative abundance of functionally annotated transcripts across samples from 2019, 2020, and 2021, classified according to COG categories. Bars represent the proportional distribution of transcripts within the categories of Information storage and processing, cellular processes and signaling, metabolism, and poorly characterized functions. Shared and unique fractions are shown separately for each sampling period. [file ECE3-15-e72786-s003.tif]

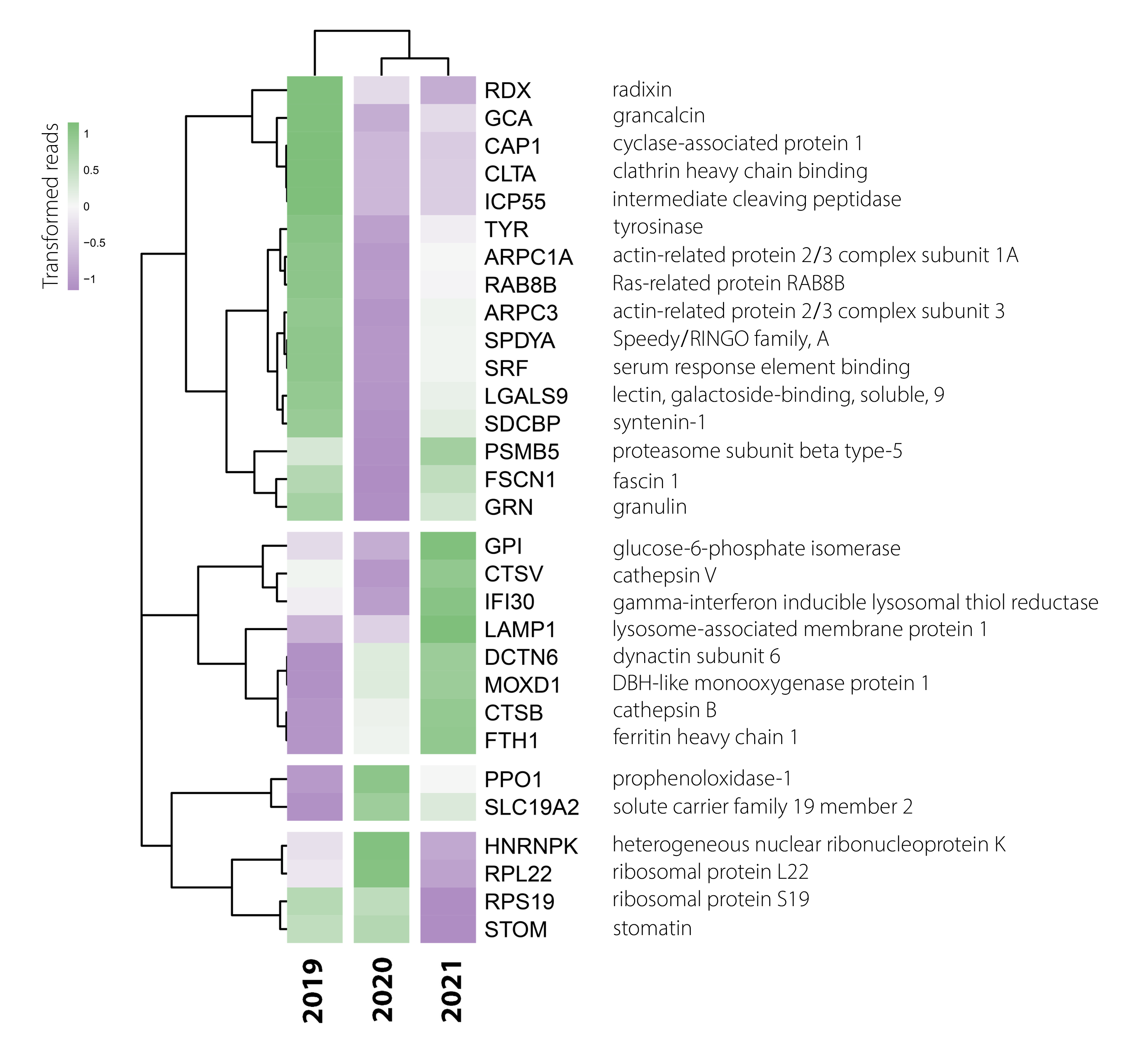

Supplement: Supplementary file 2 — Figure S2: Heatmap of Z‐score–normalized expression values for immune‐annotated transcripts. Each row represents one of the 30 transcripts categorized under the Gene Ontology term immune system process. Expression values were transformed by Log2(reads +1) and were standardized by Z‐score across transcripts to highlight relative deviations from the mean expression level for each gene. [file ECE3-15-e72786-s004.tif]
